# Amyloid inhibition by molecular chaperones *in vitro* can be translated to Alzheimer's pathology *in vivo*

**DOI:** 10.1039/d3md00040k

**Published:** 2023-03-21

**Authors:** Axel Abelein, Jan Johansson

**Affiliations:** a Department of Biosciences and Nutrition, Karolinska Institutet 141 83 Huddinge Sweden axel.abelein@ki.se

## Abstract

Molecular chaperones are important components in the cellular quality-control machinery and increasing evidence points to potential new roles for them as suppressors of amyloid formation in neurodegenerative diseases, such as Alzheimer's disease. Approaches to treat Alzheimer's disease have not yet resulted in an effective treatment, suggesting that alternative strategies may be useful. Here, we discuss new treatment approaches based on molecular chaperones that inhibit amyloid-β (Aβ) aggregation by different microscopic mechanisms of action. Molecular chaperones that specifically target secondary nucleation reactions during Aβ aggregation *in vitro* – a process closely associated with Aβ oligomer generation – have shown promising results in animal treatment studies. The inhibition of Aβ oligomer generation *in vitro* seemingly correlates with the effects of treatment, giving indirect clues about the molecular mechanisms present *in vivo*. Interestingly, recent immunotherapy advances, which have demonstrated significant improvements in clinical phase III trials, have used antibodies that selectively act against Aβ oligomer formation, supporting the notion that specific inhibition of Aβ neurotoxicity is more rewarding than reducing overall amyloid fibril formation. Hence, specific modulation of chaperone activity represents a promising new strategy for treatment of neurodegenerative disorders.

## Abstract 2

Molecular chaperones are important components in the cellular quality-control machinery and increasing evidence points to potential new roles for them as suppressors of amyloid formation in neurodegenerative diseases, such as Alzheimers disease.

## Graphical abstract

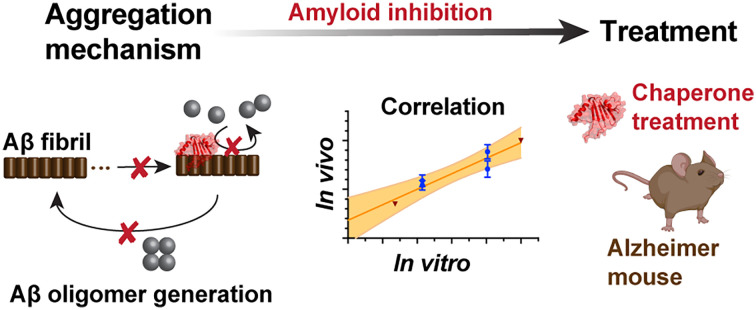

## Introduction

**Fig. 1:**
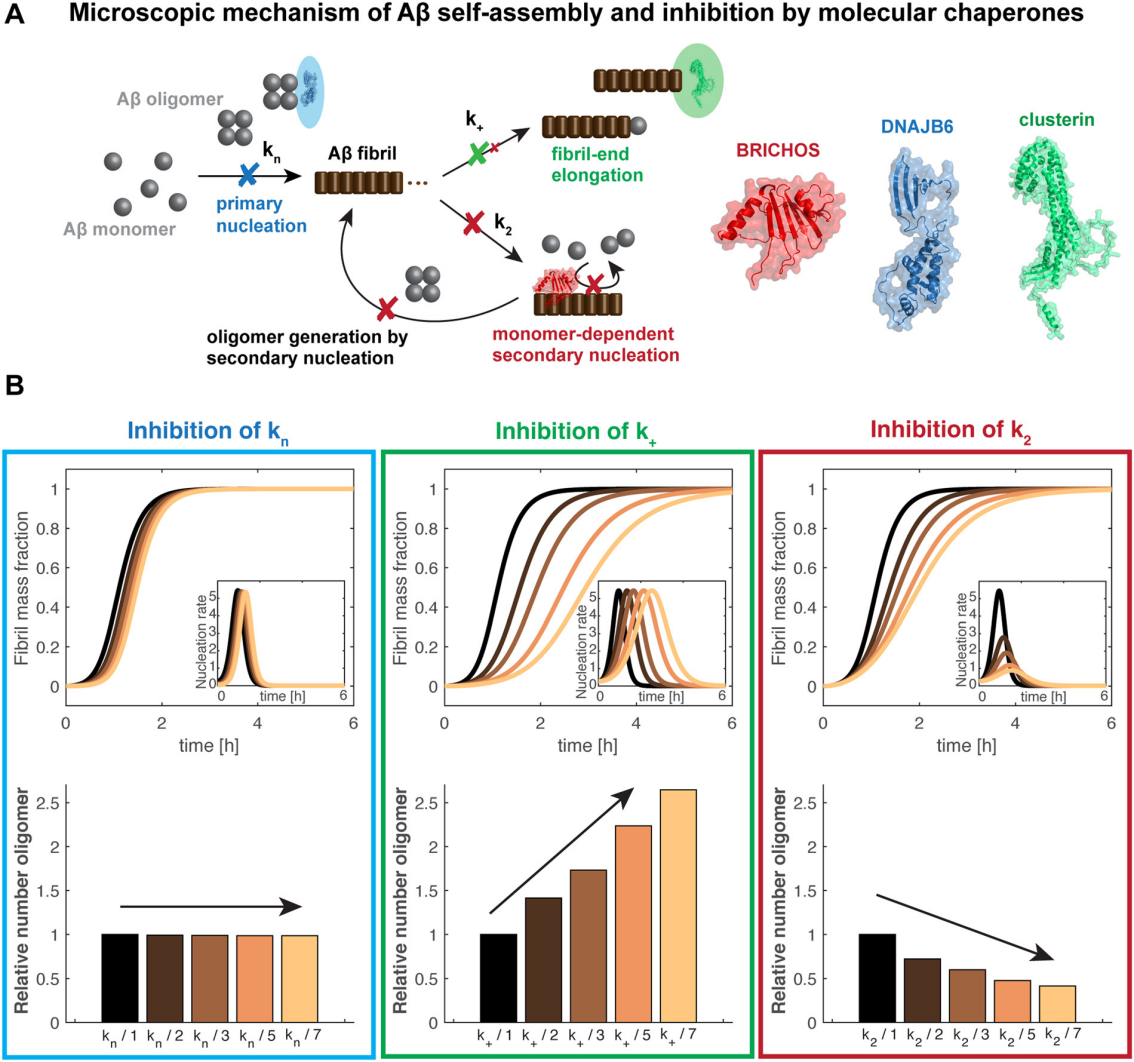
Schematic mechanism of action of chaperone-modulated inhibition of Aβ aggregation and oligomer generation *in vitro*. (A) The Aβ aggregation mechanism includes primary and secondary nucleation processes, related to the nucleation rate constants *k*_*n*_ and *k*_2_, in addition to fibril-end elongation, *k*_*+*_. Secondary nucleation is the dominant mechanism and the major source of formation of new oligomers, providing a positive feedback loop. BRICHOS mainly inhibits secondary nucleation events, preventing the generation of oligomers (here the Bri2 BRICHOS monomer is displayed).^1,3,4^ DNAJB6 was found to bind Aβ oligomers,^5^ causing a predominate reduction of primary nucleation^6,7^ (the shown structure represents a monomeric subunit of the DNAJB6 oligomer). In contrast, clusterin rather specifically prevents fibril-end elongation^11^ (here a monomeric subunit of clusterin is visualized). (B) Simulations of the generation of oligomers *in vitro* are displayed where one specific nucleation rate constant is reduced by factors 2 (dark brown), 3 (light brown), 5 (orange) and 7 (yellow), compared to non-inhibited kinetics (black). The integral of the nucleation rate over the reaction time represents the amounts of oligomers generated, shown as relative values. The kinetic parameter used for the simulations represent typical values of Aβ42 aggregation.^16^

Alzheimer's disease (AD) is the most prominent neurodegenerative disease affecting an increasing number of people worldwide due to the rising elderly society.^17^ The amyloid cascade hypothesis was put forward more than 20 years ago,^18–20^ pinpointing the misfolding and aggregation of the amyloid-β peptide as the cause of AD, preceding other characteristics such as tau pathology. Since then, many therapeutic attempts have targeted Aβ production and aggregation, either using antibody-based immunotherapies or enzyme inhibition.^21^ The main focus of these trials has been to decrease the total Aβ plaque load by inhibiting Aβ aggregation in general, or to reduce the overall Aβ production by modulating Aβ precursor protein (APP) cleavage. The drastic rate of failure of these attempts in clinical trials has led to questioning of the amyloid cascade hypothesis in general. The explanation for the lack of effective treatments might, however, rather be found in the details of the molecular mechanisms of the interventions. The fibril surface was found to play a crucial role in catalyzing the formation of new nucleation units during Aβ aggregation, in a process referred to as secondary nucleation.^16,22^ This was found to be the dominant mechanism in Aβ40 and Aβ42 (40 and 42 residue, respectively, Aβ isoforms) self-assembly, and to be the major source of the generation of presumably toxic oligomers (Fig. 1A).^16,23^ As accumulating evidence assigns pre-fibrillar oligomeric species, and not the fibril structure as such, as the most toxic species, prevention of formation of Aβ oligomers should minimize toxic effects.^17,24,25^ Targeting specific nucleation events, rather than overall aggregation, has hence been suggested as a more promising approach in the search for efficient AD therapeutics.^26^

Besides antibodies and enzyme inhibitors, recent advances *in vitro* have put focus on molecular chaperones as a natural inhibitory system against protein misfolding, aggregation and amyloid formation.^4,7,14,27^ Molecular chaperones are involved in diverse functions in the living cell and organized in an extensive network, also referred to as protein homeostasis or proteostasis.^28^ Several molecular chaperones and chaperone-like proteins have been reported to interfere also with aggregation of disease-related proteins.^28^ Such examples are the heat shock protein (HSP) DNAJB6, the extracellular chaperone clusterin and proteins of the BRICHOS domain family, which all interfere with Aβ42 aggregation. Intriguingly, chaperone mechanisms of action against Aβ42 self-assembly are substantially different.

## Inhibition of specific nucleation events modulates the Aβ oligomer generation in different ways

### The mechanism of action of Aβ self-assembly

Aβ aggregation *in vitro* can be understood as different microscopic nucleation events – primary and secondary nucleation and fibril-end elongation (Fig. 1A). Remarkably, specific inhibition of each one of these reaction steps results in differently modulated neurotoxic effects^4,14^ (Fig. 1B). The integral of the nucleation rate over the reaction time represents then the generation of new nucleation units, which can subsequently convert to Aβ oligomers. This shows that preventing secondary nucleation is accompanied by a drastic reduction of oligomeric species. In contrast, specifically attenuating primary nucleation is linked to a retarded maximum of oligomer generation, yet the overall amount of oligomers is not changed. Finally, a specific inhibition of fibril-end elongation events increases the overall amount of produced oligomers (Fig. 1B). Notably, oligomer generation can also be estimated when two or more nucleation rate constants are modulated using the general description of the nucleation rate as a function of the individual nucleation rate constants.^1,4^

The effect of aggregation modulators can be tested by conducting aggregation kinetics of Aβ at different concentrations of aggregation modulators. The fitting analysis is based on a set of master equations^29^ and can be performed using software with integrated global fit tools or the online tool Amylofit.^30^ Of importance, while the effect on the combined rate constants can be obtained from such analysis, it is more difficult to determine the effect on the individual nucleation rate constants.^30^ In such cases, experiments using a high concentration of seeds, which enable to bypass primary and secondary nucleation, can shed light on the sole effect on fibril-end elongation.^31^ These additional sets of data are highly valuable to differentiate between the nucleation constants used as combined fitting parameters and hence to deduct the specific effect on individual nucleation rate constants.

Taken together, detailed analysis of aggregation kinetics provides a forceful tool to estimate Aβ oligomer generation *in vitro*. Aggregation inhibitors that specifically target secondary nucleation and thus reduce oligomer formation are particularly interesting candidates for therapeutic interventions.^4,32^

### Molecular chaperones target different nucleation events

Many molecular chaperones and chaperone-like proteins have been reported to interfere with Aβ aggregation, including HSPs, extracellular chaperones and BRICHOS domain proteins (reviewed in ref. 33). A list of reported molecular chaperones inhibiting Aβ42 aggregation, where the effects on specific microscopic nucleation events were determined, is given in Table 1. Molecular chaperones studied in detail are DNAJB6, clusterin and the BRICHOS domain, which each inhibits predominantly one specific nucleation rate constant (Fig. 1A).

Inhibitory effect on microscopic mechanism of Aβ42 aggregation by molecular chaperones and antibodies

**Table:** 

Aggregation modulator	Targeted species of Aβ	Inhibited microscopic nucleation event	Ref.
Molecular chaperones			
BRICHOS domain			
proSP-C BRICHOS WT (trimer)	Fibril surface	2nd nucleation ↓↓	4
proSP-C BRICHOS T187R mutant (monomer)	Fibril surface & oligomers (2nd nucleation competent)	2nd nucleation ↓↓	51
Bri2 BRICHOS WT crude	Fibril surface and fibril-ends	2nd nucleation ↓↓, elongation ↓	7, 55
Bri2 BRICHOS WT monomer, dimer & oligomer	Fibril surface and fibril-ends	2nd nucleation ↓↓, elongation ↓	14
Bri2 BRICHOS R221E (monomer)	Fibril surface and fibril-ends	2nd nucleation ↓↓, elongation ↓	1

**Table:** 

Other molecular chaperones			
DNAJB6	Oligomers	Primary nucleation ↓↓	6, 7
αB-crystallin	Fibril surface and fibril-ends	2nd nucleation ↓↓, elongation ↓	7
Clusterin	Fibril-ends	Elongation ↓↓	11
S100B ± calcium	Monomers, oligomers and fibrils	2nd nucleation ↓↓, primary nucleation ↓	41
S100A9	Fibril surface	2nd nucleation ↓↓	45
Nucleobindin 1	“Pre-fibrillar species”	Primary nucleation,^a^ 2nd nucleation^a^	46
*Drosophila* and human HSP10	Possibly fibril-ends	Primary nucleation^a^ or elongation^a^	47

**Table:** 

Antibodies			
Specific single-chain antibody fragments	Fibril surface	2nd nucleation ↓↓	56
Aducanumab (murine)	Fibril surface	2nd nucleation ↓↓	15
Gantenerumab (murine)	Fibril-ends	Elongation ↓↓	15
Bapineuzumab (murine)	Fibril-ends	Elongation ↓↓	15
Solanezumab (murine)	Monomers	Primary nucleation ↓↓	15

a Only determined by fit analysis and not confirmed by seeding experiments.

DNAJB6 is a human HSP belonging to the HSP40 family and is involved in diverse processes, including protein folding. Interestingly, DNAJB6 also acts against Aβ42 aggregation with a predominate effect on primary nucleation.^6^ DNAJB6 retards Aβ42 aggregation already at low sub-stoichiometric ratios, making it one of the most efficient reported anti-amyloid chaperones. A conserved region with serine and threonine residues was identified to play an important role in preventing fibrillization by modulating primary nucleation reactions.^34,35^ DNAJB6 exists as large megadalton oligomers in equilibrium with small, dissociated subunits.^36,37^ Evidence that the chaperone binds to small pre-fibrillar Aβ species, rather than monomeric species, was provided by mass-spectrometry, suggesting that dimeric or trimeric subunits of DNAJB6 capture Aβ oligomers at different sizes.^5^ Hence, this binding of intermediate Aβ oligomers apparently causes the reduction of the primary nucleation rate.

Clusterin, aka apolipoprotein J, belongs to a family of extracellular chaperones and its molecular mechanism of interaction with Aβ42 was assigned to prevention of fibril-end elongation events.^11^ Clusterin can bind to different aggregation states of Aβ including oligomers and mature fibrils,^11,38,39^ and clusterin itself exists in a range of different oligomeric forms.^40^ Notably, while a first global fit analysis using the combined rate constants^39^ concluded a modulation of both primary and secondary Aβ42 nucleation by clusterin, the addition of highly seeded experiments enabled the authors of a subsequent study to explain the observed effect as an individual effect on elongation,^11^ highlighting the value of seeded data sets.

Another example of an amyloid-suppressing protein is the proinflammatory protein S100B. Calcium binding modulates its function and structure, where in particular the calcium-bound state binds Aβ monomers.^41^ This interaction leads to inhibition of primary nucleation, but S100B was also shown to bind to Aβ fibrils and to inhibit secondary nucleation, which is enhanced by the presence of calcium.^41^ S100B is hence an example for an aggregation-inhibiting protein that influences several nucleation reactions.

Another example of a molecular chaperone affecting two nucleation events is αB-crystallin, inhibiting elongation and secondary nucleation.^7^ αB-crystallin is an ATP-independent small HSP and forms large polydisperse complexes with varying number of subunits.^42,43^ Different sites were identified to bind either Aβ fibrils or amorphous clients.^44^ The binding interface apparently exhibits such structural plasticity that αB-crystallin target both the Aβ fibril surface and the fibril-ends.

Also the proinflammatory S100A9 protein affects different nucleation events, with a dominant inhibitory effect on secondary nucleation.^45^ Yet other molecular chaperones for which specific effects on the microscopic nucleation process of Aβ42 have been found include nucleobindin 1 (ref. 46) and *Drosophila* and human HSP10.^47^ Here, multiple effects on Aβ fibrillation kinetics were reported and a clear distinction could not be made, possibly due to the lack of additional seeding experiments.

The BRICHOS domain protein family has been studied in detail.^1,4,14^ The BRICHOS domain is found in 13 different families and associated to different diseases.^3,48,49^ Amyloidogenic peptides can be released from proproteins in which the suggested primary function of BRICHOS is to prevent the potentially amyloid-forming polypeptides from aggregation.^49^ The two most studied examples are Bri2 BRICHOS, which is found in dementia-associated integral membrane protein 2B (ITM2B aka Bri2) and BRICHOS from prosurfactant protein C (proSP-C), which is implicated in interstitial lung disease.^48–50^ proSP-C BRICHOS exists predominately as a trimer and inhibits Aβ42 fibrillization by binding to the fibril surface and preventing secondary nucleation events.^4^ Studies using a single-point mutant of proSP-C BRICHOS, which favors the monomeric state, showed that also this variant predominately inhibits secondary nucleation and captures the smallest secondary nucleation-competent Aβ oligomers.^51^

Bri2 BRICHOS prevails in three different assembly states (monomers, dimers and oligomers), which were found to execute specific functions.^14^ The Bri2 BRICHOS monomers and dimers were most efficient in inhibition of Aβ42 fibrillization and in particular the monomers could drastically reduce Aβ42-associated neurotoxic effects.^14^ The Bri2 BRICHOS oligomers, in contrast, act more as classical chaperones, preventing most effectively amorphous aggregation of client proteins.^14,52^ The mechanism of action to retard Aβ42 fibril formation was assigned to inhibition of secondary nucleation in addition to fibril-end elongation.^14^ A single-point mutant (R221E) that stabilizes the monomeric state of Bri2 BRICHOS, has shown similar effects as the Bri2 BRICHOS monomer, inhibiting the same microscopic nucleation events.^1^ Important in the context of future development as drug candidates, the Bri2 BRICHOS monomer was shown to pass the blood–brain in mice.^53,54^

Taken together, several molecular chaperones have shown promising results *in vitro*, strongly preventing Aβ aggregation and/or selectively targeting secondary nucleation and preventing oligomer generation, which encouraged studies to test their effect *in vivo*.

## Translation of aggregation inhibitory effects by molecular chaperones to the *in vivo* situation

### Simple model systems exhibit reduction of Aβ-associated toxicity

The effect on Aβ-associated toxicity by molecular chaperones (with an identified effect on microscopic aggregation mechanism) has been tested as impact on viability of neuronal cells^41^ and γ-oscillations in mouse hippocampal slices,^1,4,14,55^ and could be studied in *Caenorhabditis elegans* models as performed for other amyloidogenic proteins.^27^ For the studied S100B and different BRICHOS domain proteins, which all target secondary nucleation processes, a suppression of toxicity was reported, confirming the effect observed *in vitro*.

Further, *Drosophila* models were used where transgenic co-expression of BRICHOS and Aβ42 in the central nervous system was found to prevent Aβ42-associated toxicity, measured as improved longevity and locomotor activity.^55,57^ Interestingly, BRICHOS co-localized with Aβ42 amyloid plaques in the brain of the flies and improved the eye phenotypes compared to Aβ42 expressing flies.^55^ Hence, the BRICHOS domain has been shown to prevent Aβ42-associated toxic impact in simple *in vivo* model systems, where the observed positive effects motivated to continue with more advanced model systems.

### Bri2 BRICHOS reduces neuroinflammation and improves cognitive behavior in AD mouse models

**Fig. 2:**
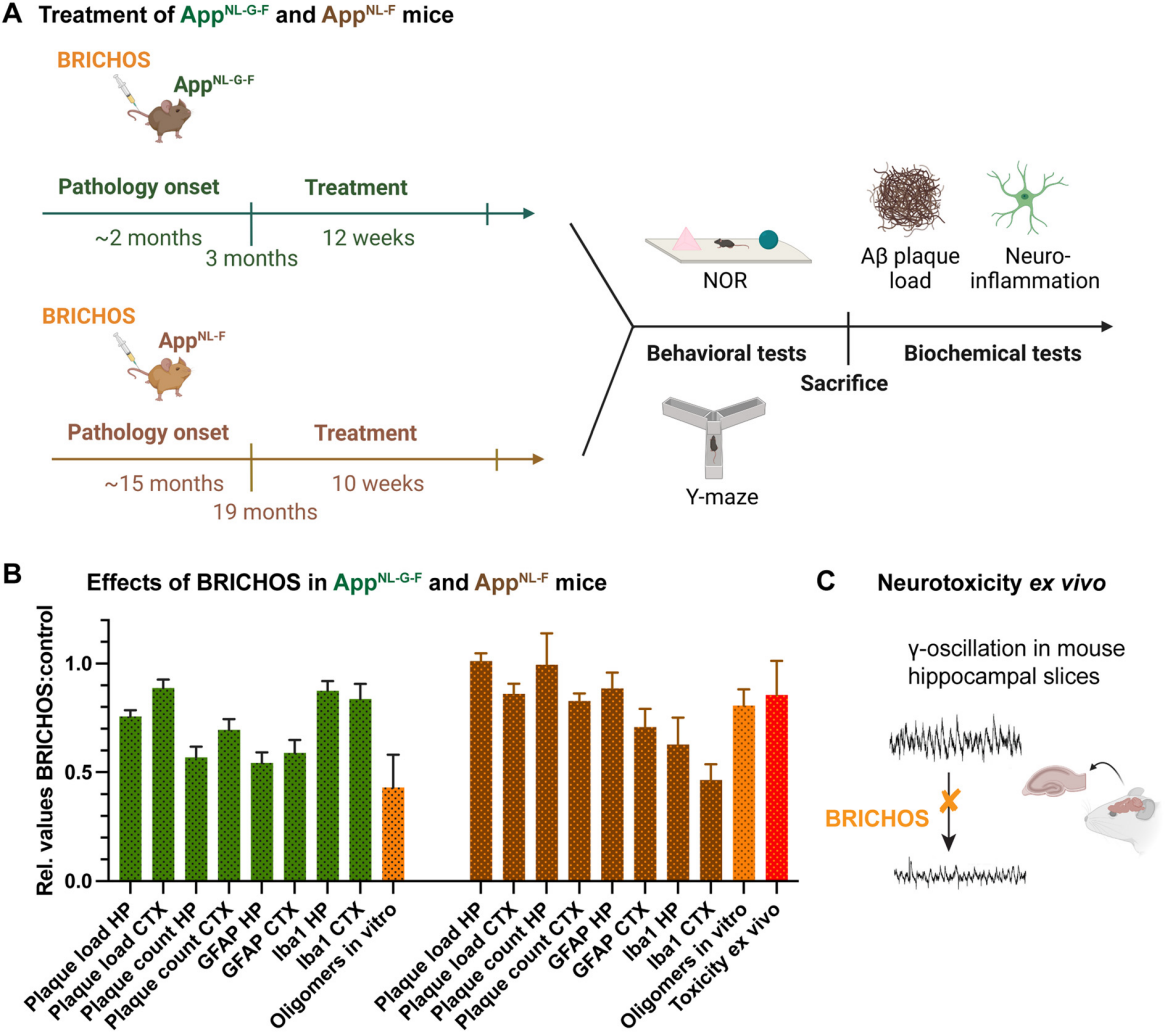
Effects of Bri2 BRICHOS in AD mice. (A) Two different AD mouse models, App^NL-G-F^ and App^NL-F^, were treated with repeated intravenous injections of the R221E Bri2 BRICHOS monomer mutant,^1^ where the treatment start was chosen to be around AD pathology onset for App^NL-G-F^ and after AD pathology onset for App^NL-F^ mice, respectively.^2^ After treatment the mice were subjected to behavioral test novel object recognition (NOR) and Y-maze and after sacrifice biochemical analyses of plaque count/load and neuroinflammation were performed.^2^ (B) The relative effects on biochemical parameters in cortex (CTX) and hippocampus (HP) are displayed for the BRICHOS treated against the control group. Further, the reductions of Aβ42 oligomer generation from *in vitro* experiments are shown.^1,9^ (C) The impact of BRICHOS on Aβ42-associated neurotoxicity was measured as reduction of γ-oscillations in mouse hippocampal slices.^1,14^

A recent study of BRICHOS in AD mouse models showed treatment effects, including reduction of neuroinflammation and improvement of cogitative behavior^2^ (Fig. 2A). Two different APP knock-in mouse models were given repeated intravenous injections of monomeric R221E Bri2 BRICHOS.^1^ One model, referred to as App^NL-F^, harbors the Swedish and Beyreuther/Iberian mutations (located outside the Aβ stretch in the App sequence), leading to enhanced Aβ production and increased Aβ42 : Aβ40 ratio.^58^ This model develops plaque pathology, astrogliosis and microgliosis from an age of 9–12 months.^58^ The second model, referred to as App^NL-G-F^, carries additionally the Arctic mutation (E22G), which is located within the Aβ sequence and produces the aggregation-prone Arctic Aβ42.^59^ The App^NL-G-F^ model is distinguished by rapid development of AD-like pathology already from an onset at 2–4 months.^58^ The App^NL-F^ model was treated at an age of 19 months, *i.e.* within a time window when AD pathology had already been established. In contrast, for the App^NL-G-F^ the treatment was started at an age of 3 months, *i.e.* coinciding with the start of developing AD pathology.

Both treatments resulted in reduction of plaque burden and attenuated neuroinflammation as indicated by the astrocyte marker glial fibrillary acidic protein (GFAP) and microglial activation marker ionized calcium-binding adapter molecule 1 (Iba1), with more pronounced effects for the App^NL-G-F^ model (Fig. 2B). Plaque burden was analyzed by thioflavin S or 82E1 Aβ antibody staining, referred to as plaque count and plaque load, respectively. Measuring the effects on cognitive behavior revealed significant improvements in learning and memory for the App^NL-G-F^ but not for the App^NL-F^ model. In conclusion, these findings represent the first AD treatment study using intravenous administration of a molecular chaperone domain, demonstrating cognitive improvement when treatment was initiated at an early stage and positive effects on neuroinflammation when treatment was initiated at early and advanced stages of AD pathology.

### Inhibition of Aβ42 oligomer generation *in vitro* by BRICHOS correlates with improved Alzheimer's pathology *in vivo*

Due to the predominant inhibition of the secondary nucleation events by monomeric R221E Bri2 BRICHOS mutant an inhibition of oligomer generation is expected *in vitro* (Fig. 1). Indeed, calculating the generation of new nucleation units from aggregation kinetics data revealed a reduction of ∼70% of oligomers at a 1 : 1 BRICHOS : Aβ42 ratio.^1^ Importantly, R221E Bri2 BRICHOS delays the aggregation of Artic Aβ42 similarly to WT Aβ42, by specifically reducing the secondary nucleation rate constant, leading to a reduction of oligomer generation also for Artic Aβ42.^9^

**Fig. 3:**
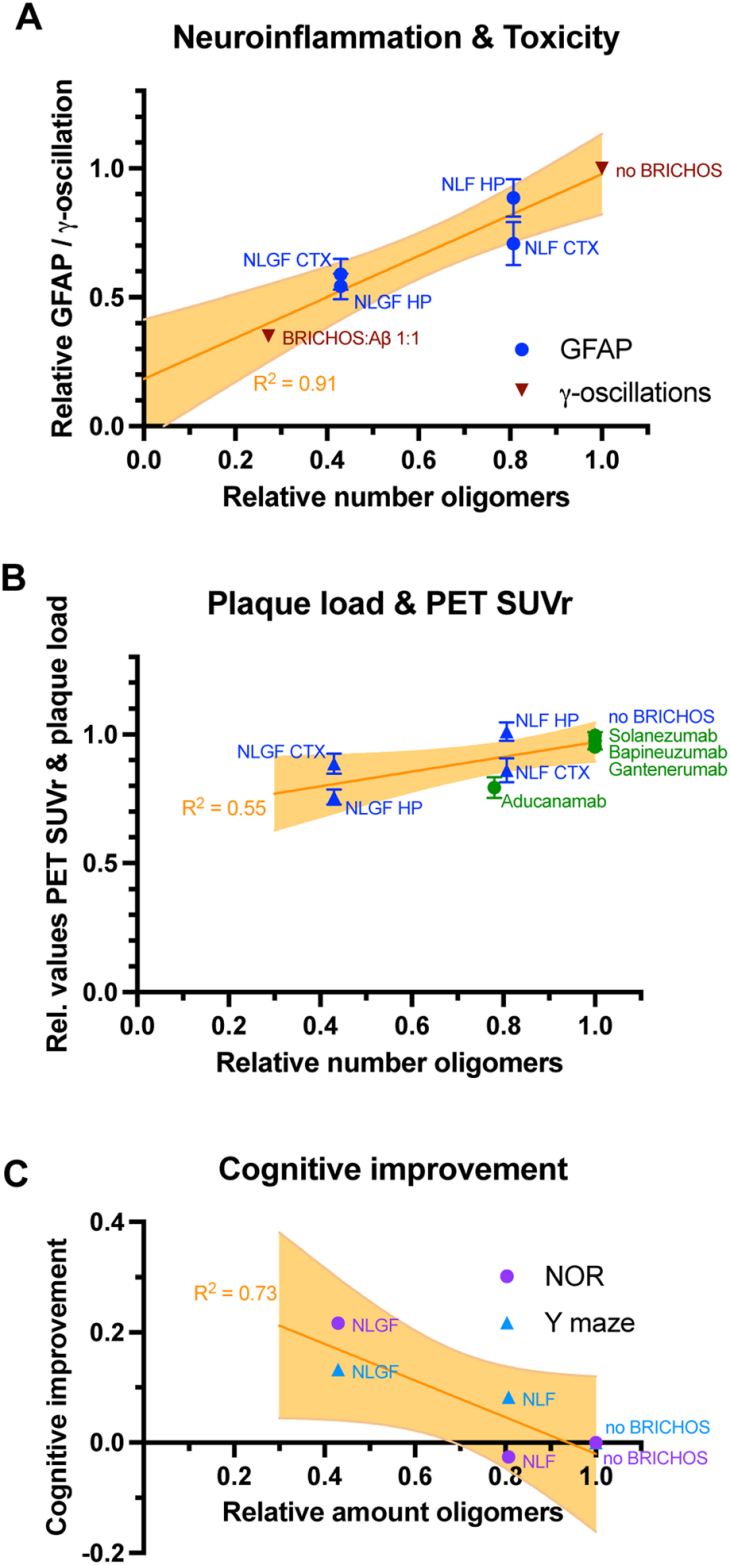
Correlations of Aβ42 oligomer generation *in vitro* with *in vivo* treatment effects. (A) Levels of the neuroinflammation marker GFAP upon Bri2 BRICHOS treatment, measured in the hippocampus (HP) and cortex (CTX) of App^NL-G-F^ (NLGF) and App^NL-F^ (NLF) mice,^2^ and toxicity *ex vivo*, obtained as γ-oscillations in mouse hippocampal slices^1^ correlate with Aβ42 oligomer generation *in vitro*.^1^ (B) The reduction of Aβ plaque load of both AD mouse models after Bri2 BRICHOS treatment^2^ exhibits only a weak correlation with the reduction of Aβ42 oligomer *in vitro* by Bri2 BRICHOS.^1^ Published values of the reduction of PET SUVr values by the antibodies aducanumab,^8^ gantenerumab,^10^ bapineuzumab^12^ and solanezumab^13^ can be related to their effects on secondary nucleation.^15^ This relation apparently follows a similar trend as observed in the BRICHOS treatment studies of AD mice.^2^ (C) Cognitive improvement of AD mice after BRICHOS treatment, measured by novel object recognition (NOR) and Y maze experiments, exhibits a trend for correlation with the impact of BRICHOS on Aβ42 oligomer generation *in vitro*.

An interesting question is then whether the effects seen *in vitro* can be quantitatively translated to the effects observed in treatment studies of AD mice. To address this question the ratio of BRICHOS : Aβ42 in the brain can be estimated based on the extent of BBB passage of BRICHOS^53^ and the measured Aβ42 levels in the brain.^2^ Using this BRICHOS : Aβ42 ratio the relative generation of oligomers can be estimated from the corresponding aggregation kinetics data *in vitro*.^1,9^ These estimations reveal a decrease in generation of oligomers to 81 ± 7% and 43 ± 15% compared to the original values without BRICHOS for WT and Arctic Aβ42, respectively (Fig. 2B). The relative GFAP values in App^NL-G-F^ and App^NL-F^ hippocampus and cortex, between the treated and control groups can then be related to the relative number of oligomers estimated from *in vitro* results (Fig. 3A). Similarly, the effect of BRICHOS on the γ-oscillation measured in mouse hippocampal slices (Fig. 2C) can be related to the generation of nucleation units *in vitro* at a given BRICHOS : Aβ42 ratio. Plotting the relative effects on GFAP levels *in vivo* and γ-oscillation impact *ex vivo* against the relative number of oligomers reveals a strong correlation (*R*^2^ = 0.91, *p* = 0.003, Fig. 3A). In contrast, correlating the amount of oligomers with the plaque load only results in a very weak correlation (*R*^2^ = 0.55, *p* = 0.15, Fig. 3B). Also relative improvements in cognitive function measured by novel object recognition (NOR) and Y maze tests, which are related to learning and memory, can be correlated with oligomer formation *in vitro*. Interestingly, here a moderate correlation is obtained (*R*^2^ = 0.73, *p* = 0.06, Fig. 3C), yet due to the few data points should be interpreted as a trend.

Hence, the reduction of oligomer generation determined *in vitro* strongly correlates with attenuated levels of neuroinflammation marker GFAP *in vivo* and seemingly also correlates with improvements of cognitive behavior after BRICHOS treatment. There is however no significant correlation between oligomer formation *in vitro* and observed plaque load *in vivo*, even though an overall reduction of plaque amount was evident from the *in vivo* data. Notably, it has been shown previously that producing Aβ42 and BRICHOS at equimolar amounts from a common precursor in transgenic mice results in unaltered plaque load but markedly reduced oligomer formation and no cognitive decline compared to when Aβ42 is produced form APP without any BRICHOS overexpression.^60^ Also, transgenic overexpression of BRICHOS in APP/presenilin 1 mutant mice results in modest reduction in plaque load but marked reduction in GFAP levels and improved cognition.^61^

## Antibodies specifically targeting Aβ oligomer generation show positive effects in clinical trials

Several antibodies have been or are currently in AD clinical trials. The antibodies gantenerumab,^10^ bapineuzumab^12^ and solanezumab^13^ have been investigated in clinical phase IIb and III trials, yet no significant improvements in clinical symptoms were observed, as reviewed in ref. 62. However, last year (2021), the antibody aducanumab (Biogen) was approved by the FDA for treatment of Alzheimer's patients, making it the first approved Alzheimer drug since decades. Administration of aducanumab gave significant reduction in the Aβ plaque load in patients with prodromal or mild AD measured by positron emission tomography (PET) imaging, reported as standard uptake value ratio (SUVr).^8^ Further signs of improvement were reported for specific groups of patients, yet the overall behavioral analysis showed only mild improvements, resulting in a debate about the FDA approval of the drug.^63^ A recent study investigated the molecular mechanism of the murine analogs of aducanumab, gantenerumab, bapineuzumab and solanezumab, with respect to their ability to inhibit secondary nucleation and oligomer generation.^15^ While all antibodies reduced the overall aggregation, they exhibited very different mechanisms of action in inhibiting Aβ42 self-assembly. The murine versions of gantenerumab and bapineuzumab preferably bound fibrillar species and specifically interfered with elongation of monomers to the fibril-ends. On the contrary, the murine analog of solanezumab had a high binding affinity to monomeric Aβ and prevented primary nucleation events. Murine aducanumab exhibited a high preference towards fibrils compared to monomers, and specifically inhibited secondary nucleation processes. Since secondary nucleation is closely associated to oligomer formation for Aβ42 aggregation (Fig. 1), aducanumab was the only antibody that attenuated oligomer generation.^15^ Interestingly, plotting the relative amounts of PET SUVr against their ability to reduce secondary nucleation events *in vitro*, approximately the same trend is followed as observed by the effect of BRICHOS on the plaque load in AD mice (Fig. 3B).

Very recently (Sept 2022), another antibody, lecanemab, has successfully met primary endpoints in a clinical phase III study.^64^ Lecanemab is a monoclonal antibody that was generated to specifically bind proto-fibrils, defined as small fibrillar species preceding mature fibril structures, which exhibit high neurotoxicity.^65^ Cognitive improvement, as measured by a global cognitive and functional scale (CDR-SB) was reported to 27% compared with placebo at 18 months.^64^ Hence, the two antibodies that present successful clinical phase III results exhibit abilities to bind small Aβ42 aggregates and/or modulate Aβ42 oligomer generation.

## Conclusions and outlook

The specific effects of several selected molecular chaperones on Aβ self-assembly have been tested *in vitro*, but so far only a limited number of chaperones have also been investigated in living model systems, determining their ability to suppress Aβ-associated toxicity. Considering the large number of chaperones in the proteome it is likely that an increasing number will be evaluated in the future. The most promising candidates in *in vitro* and *ex vivo* toxicity studies show specific effects on secondary nucleation and Aβ oligomer formation. Recent results for the BRICHOS domain show an apparent correlation between the inhibitory effect on Aβ oligomer generation *in vitro* and attenuation of the levels of neuroinflammation markers, as well as a trend towards quantitatively related cognitive improvement *in vivo*. Together with the observation that the Aβ plaque load of AD mice is less affected upon treatment with the molecular chaperone BRICHOS, these results indicate that inhibition of specific microscopic mechanisms is more promising than suppression of overall amyloid generation. The improved performance of antibodies that specifically target the generation of Aβ oligomers in recent immunotherapy studies gives additional hope that rationally designed approach to combat AD and other related neurodegenerative disease will give positive results.

## Author contributions

A. A. and J. J. analysed the data and wrote the article.

## Conflicts of interest

There are no conflicts to declare.

## Supplementary Material
